# Recurrent fever of unknown origin: An overlooked symptom of Fabry disease

**DOI:** 10.1002/mgg3.1454

**Published:** 2020-08-14

**Authors:** Yi Luo, Di Wu, Min Shen

**Affiliations:** ^1^ Department of Rheumatology Peking Union Medical College Hospital Chinese Academy of Medical Sciences & Peking Union Medical College National Clinical Research Center for Dermatologic and Immunologic Diseases (NCRC‐DID Key Laboratory of Rheumatology and Clinical Immunology Ministry of Education Beijing China

**Keywords:** Fabry disease, fever, fever of unknown origin

## Abstract

**Objective:**

Fabry disease (FD) is a rare X‐linked lysosomal storage disorder due to the absent or deficient activity of lysosomal hydrolase a‐galactosidase A (α‐Gal A), which leads to the accumulation of its substrates in various organs and tissues. Classic clinical manifestations include angiokeratomas, proteinuria, renal failure, neuropathic pain, and left ventricular hypertrophy. Fever is one of the rare symptoms that may occur during FD.

**Methods:**

Three Chinese Han patients with FD referred to Peking Union Medical College Hospital were reported. The complete medical records were established, and detailed data were collected. Whole‐exome sequencing by next‐generation sequencing and α‐Gal A enzyme activity assay were performed to confirm the diagnosis.

**Results:**

These three patients all presented with recurrent fever of unknown origin initially, accompanied with arthralgia/arthritis and other symptoms. We identified two known variants in the *GLA* gene, c.1176_1179delGAAG and c.782G>A (p.G261D), and a novel variant c.440G>A (p.G147E) which is likely pathogenic in our patient.

**Conclusions:**

FD should be considered as a rare cause of recurrent fever of unknown origin. The coexistence of gene variants related to systemic autoinflammatory diseases may make the clinical phenotypes of FD more complex and prone to recurrent fever.

## Graphical abstract

This is the first case series report of Chinese Fabry disease (FD) patients in whom recurrent fever of unknown origin was the initial manifestation. Two known variants in the GLA gene, c.1176_1179delGAAG and c.782G>A (p.G261D), and a novel variant c.440G>A (p.G147E) which is likely pathogenic in our patient were detected. We conclude that FD should be considered as a rare cause of recurrent fever of unknown origin.

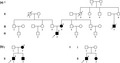

## 1 INTRODUCTION

Fabry disease (FD, OMIM: 301500) is a rare X‐linked lysosomal storage disorder due to the absent or deficient activity of lysosomal hydrolase a‐galactosidase A (α‐Gal A), which is initially described in 1898 (Anderson, 1898; Fabry, 1970; Germain, 2010). α‐Gal A is encoded by the *GLA* gene (OMIM: 300644) located on the long arm of the X Chromosome (Xq22.1), and more than 500 *GLA* variants have been reported (Nagueh, 2014). Despite the X‐linked inheritance pattern, FD affects both men and women. However, female patients are generally less frequently and less severely affected with a later onset than male patients, probably because of X‐chromosome inactivation (Echevarria et al., 2016; Hopkin et al., 2008; Wilcox et al., 2008). The exact incidence of FD is unclear, and it has been estimated at about 1/3100‐1/170000 worldwide, which may have been underestimated since most of the FD complications are nonspecific (Hwu et al., 2009; Inoue et al., 2013; Schiffmann & Ries, 2016; Spada et al., 2006). There are several reasons for the wide range of the incidence, such as regional differences in new born screening to detect mutations of doubtful significance, common mutations in certain populations, and the impact of late onset‐attenuated forms found in population screening.

The defect of α‐Gal A causes metabolism disturbances, which lead to intracellular accumulation of glycosphingolipids with terminal a‐D‐galactosyl residue, mainly globotriaosylceramide (GL‐3) , as well as globotriaosylsphingosine (lyso‐GL‐3) and galabiosylceramide (Ga‐2). These lipids accumulate in many organs and tissues, especially the vascular endothelium, kidney, heart, and nervous system. During childhood and adolescence (≤16 years), patients are mainly characterized by acroparesthesia/acrodynia, hypohidrosis/anhidrosis, and angiokeratomas. The severe pain crisis can be accompanied by fever. Sensorineural hearing loss and nonspecific gastrointestinal symptoms may also appear. During early adulthood (17–30 years), acroparesthesia/acrodynia could improve with more extensive angiokeratomas, and then, ophthalmologic abnormalities, headache, stomachache, proteinuria, and progressive renal insufficiency develop. Some patients manifest cardiac hypertrophy, conduction block, and valvular disease. End‐stage renal disease and life‐threatening cardiovascular or cerebrovascular complications are the most common causes of death. In the absence of universal guidelines, the diagnosis is mainly based on clinical manifestations and confirmed by pathological examination, α‐Gal A activity detection, and the *GLA* gene testing (Germain, 2010; Schiffmann et al., 2017; Zarate & Hopkin, 2008).

Timely diagnosis and early treatment are favorable for disease prognosis. However, owing to the overlap of its clinical spectrum with other diseases, FD often has a delayed diagnosis or misdiagnosis. Recurrent fever represents a rare manifestation of FD, yet is easily overlooked (Chao, Yang, & Kao, 2012; Laney et al., 2015; Lidove et al., 2012; Marchesoni et al., 2010). Herein, we aimed to describe the features of FD patients presenting with recurrent fever.

## 2 PATIENTS AND METHODS

Three Chinese Han patients with FD referred to our tertiary medical center were identified and included in this study. The complete medical records were established, and detailed data were collected. This study was approved by the Institutional Review Board of Peking Union Medical College Hospital and performed according to the Declaration of Helsinki. Informed consents were obtained from all participants. The activity of α‐Gal A was detected by fluorophotometry (Joy Orient Translational Medicine Research Centre Co., Ltd, Beijing, China). Whole‐exome sequencing by next‐generation sequencing was performed in the Center for Genetic Testing, Joy Orient Translational Medicine Research Centre Co., Ltd, Beijing, China.

## 3 CASE REPORT

### 3.1 Patient 1

**Figure 1:**
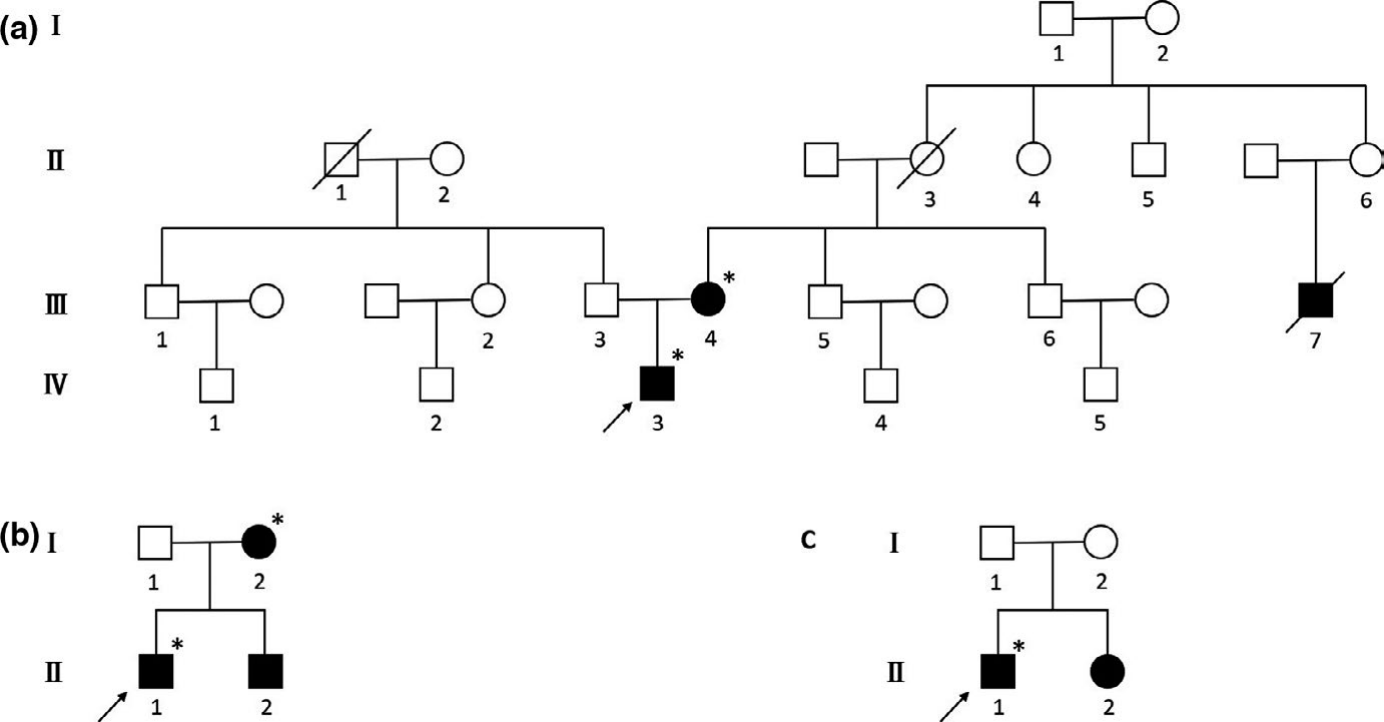
Pedigrees of Three Chinese Han Patients. (a) Pedigree of patient 1. The asterisks indicate the individuals who carry the *GLA* c.440G>A, p.G147E variant (NM_000169). (b) Pedigree of patient 2. The asterisks indicate the individuals who carry the *GLA* c.1176_1179delGAAG variant (NM_000169). (c) Pedigree of patient 3. The asterisks indicate the individuals who carry the *GLA* c.782G>A, p.G261D variant (NM_000169). The arrows indicate the probands. The individuals who had clinical manifestations are marked in black

The patient was a 15‐year‐old Chinese boy suffered from acrodynia and intermittent fever for 7 years. The pain of toes appeared at the age of 8, followed by the ache of fingers. The symptom was accompanied by recurrent low‐grade fever and hypohidrosis. The attacks were usually triggered by stress or physical exercise, and were aggravated by hot compress, with each episode lasting several days once every several months. He also had heat and cold intolerance. The pain was so severe that he could not study, exercise, or even have a rest during the episodes. He denied rash, arthralgia/arthritis, ophthalmitis, chest pain, abdominal pain, myalgia, headache, or other symptoms. His father was asymptomatic. His mother also complained of limb pain and hypohidrosis, but the symptoms were milder. An uncle on his mother's side also had foot pain and died of unknown cause in his forties (Figure 1a).

Laboratory tests showed that the full blood count (FBC), liver and renal function panels, and urine analysis were all within normal range. Erythrocyte sedimentation rate (ESR) and C‐reactive protein (CRP) were normal. Serological markers for systemic autoimmune diseases, including antinuclear antibodies (ANAs), anti‐neutrophil cytoplasmic antibodies (ANCA), and rheumatoid factor were all negative. No abnormalities were found on echocardiography. The activity of α‐Gal A was 1.8 nmol/mg/h (normal range: 24.5–86.1 nmol/mg/h), suggesting functional defect. Genetic testing revealed a hemizygous mutation in exon 3 of the *GLA* gene, c.440G>A, which led to a substitution of glutamic acid for glycine (p.G147E, NM_000169). His mother carried the same heterozygous *GLA* G147E mutation. Another two gene variants associated with autoinflammatory diseases were also identified, including a maternal heterozygous *NLRC4* p.S522P (c.1564T>C, exon 4, NM_021209), and a paternal heterozygous *NOD2* p.S30L (c.89C>T, exon 2, NM_022162). He had no response to colchicine treatment (0.5 mg, three times a day). Based on the above‐mentioned clinical manifestations and test results, the diagnosis of FD was established.

### 3.2 Patient 2

A 28‐year‐old Chinese man presented with recurrent fever and arthralgia for 17 years. The febrile attacks occurred every 4 weeks and each episode lasted 1–2 days, which was associated with a high‐temperature environment. Fever could be relieved by antipyretics. He also had arthralgia involving small joints of fingers and toes without swelling, obvious fatigue, and hypohidrosis during the febrile episodes. He additionally experienced diarrhea and chest pain. He noted hearing loss of his right ear when he was 24. He had no manifestation of eyes except for myopia. There was no numbness of limbs, angiokeratomas, rash, oral ulcer, myalgia, or headache. Pedigree analysis identified both his brother and mother with recurrent fever. His father had no symptoms (Figure 1b).

Laboratory investigation showed normal FBC, urinalysis, liver and renal function panels, ESR, and CRP. Serological markers for systemic autoimmune diseases, including ANAs, ANCA, and rheumatoid factor were all negative. Echocardiography revealed mild mitral and tricuspid valve regurgitation. Serum level of α‐Gal A enzyme activity was markedly reduced (1.1 nmol/mg/h). Gene testing revealed a hemizygous mutation in exon 7 of the *GLA* gene, c.1176_1179delGAAG, p.R392Sfs*2 (NM_000169), in the proband and the same heterozygous *GLA* gene mutation in his mother. Due to personal reasons, his brother did not receive the gene testing. In addition, the proband had a paternal heterozygous *MEFV* gene variant R202Q (c.605G>A, exon 2), though he had no response to colchicine 0.5 mg, two times a day.

### 3.3 Patient 3

A 17‐years‐old Chinese boy was referred to our hospital because of progressively worsening pain in the feet and hands accompanied by recurrent fever for 10 years. At the age of 7, he noticed intermittent pain in bilateral toes accompanied by local redness and swelling, without obvious aggravating and relieving factors. The pain episodes always complicated by fever, occasionally with chills. He developed arthralgia with bilateral metatarsophalangeal, knee, and interphalangeal joint involvement from the age of 13, accompanied with myalgia. Ultrasound showed joint effusion in the knees. As the condition continued to deteriorate, the pain attacks made him unable to sleep and contributed to high blood pressure. He felt prominent fatigue and had superficial lymphadenopathy. There was no headache, dizziness, rash, abdominal pain, or diarrhea. He had been successively treated with non‐steroidal anti‐inflammatory drugs (NSAIDs), methotrexate, leflunomide, thalidomide, colchicine, and etanercept, but without effect. Pedigree analysis revealed his sister with intermittent pain in fingers and toes from the age of 13 (Figure 1c).

Laboratory investigation including FBC, routine urinalysis, liver and renal function panels, ESR, and CRP were normal. Serological markers for systemic autoimmune diseases were all negative. Echocardiography showed posterior tricuspid valves prolapse and mild tricuspid insufficiency. A 24‐hour dynamic electrocardiogram indicated sinus arrhythmia and occasional ventricular premature contractions. Serum level of α‐Gal A activity was close to zero (0.2 nmol/mg/h), confirming the deficiency of α‐Gal A. A hemizygous mutation in exon 5 of the *GLA* gene (c.782G>A, p.G261D, NM_000169) was identified, which was likely pathogenic. His family did not receive genetic analysis.

## 4 DISCUSSION

Combining the clinical manifestations, family histories, serum activity ofα‐Gal A, and identification of the *GLA* gene mutations, these three patients could be definitely diagnosed as FD. In this study, we identified two variants in the *GLA* gene which have been found to be pathogenic or likely pathogenic in FD. A four‐base deletion c.1176_1179delGAAG in exon 7 was found in patient 2, which resulted in a frameshift in codon 392, the incorporation of threonine and premature termination after codon 394, and was proved to be a pathogenic variant (Topaloglu et al., 1999). Another G‐to‐A transition of in codon 261 of exon 5 (c.782G>A), resulting in the replacement of glycine with aspartic acid (G261D), was identified in patient 3. G261D variant was reported to be pathogenic and predicted to be probably damaging on protein function (Altarescu et al., 2001; Ashley, Shabbeer, Yasuda, Eng, & Desnick, 2001; Branton et al., 2002; Shin et al., 2008; Takata et al., 1997). Simultaneously, we identified a novel variant in the *GLA* gene in patient 1 and his mother, G147E (c.440G>A), which is characterized by a G/A substitution in exon 3, resulting in the exchange of glycine to glutamic acid. Although this variant has been reported neither in the literature nor mutation databases of FD (http://fabry‐database.org/), it was classified as “likely pathogenic” according to ACMG (American College of Medical Genetics and Genomics) guidelines. Therefore, we consider G147E as a novel variant which is likely pathogenic in FD, but the accurate significance was unknown.

Intriguingly, we found that recurrent fever was a presenting feature of all three patients in our study. As we know, this is the first case series report of Chinese FD patients in whom recurrent fever of unknown origin (RFUO) was the initial manifestation. As regard to RFUO, the differential diagnosis is always a big challenge to physicians. RFUO is characterized by repetitive febrile episodes which last a few days or a few weeks, and are separated by symptom‐free intervals of varying duration (Knockaert, Vanderschueren, & Blockmans, 2003). Possible causes of RFUO include infections, neoplasms, non‐infectious inflammatory diseases, and a miscellaneous group of more than 200 different diseases. The most relevant rare diseases associated to RFUO encompass systemic autoinflammatory diseases (SAIDs), for which genetic tests are usually required (including mutations in the following genes: *MEFV*, *TNFRSF1A*, *MVK*, *NLRP3*, *NOD2*, etc.), and lysosomal storage disorders (LSDs) (including FD, Gaucher disease, Pompe disease, mucopolysaccharidoses, etc.), for which specific enzyme assays and genetic tests are required. Studies have shown that FD may present itself with RFUO (Abreo, Oberley, Gilbert, Opitz, & Updike, 1984; Dinc, Simsek, Pay, Caglar, & Can, 2000; el‐Shahawy, Mesa, Koss, & Campese, 1996; Kikumoto et al., 2010; Sawada et al., 2015; Zizzo et al., 2013). An International Panel for Rare Recurrent FUO (IPRAFUO) recommended the inclusion of FD as a rare febrile condition in existing diagnostic algorithms for fever of unknown origin (Manna et al., 2017). A retrospective analysis demonstrated that 20.7% patients with FD presented with fever at the onset of the disease, and 83% of patients with fever also exhibited acroparesthesia. They also found the absence of a significant increase of systemic inflammation markers during fever and a weak response of fevers to common antipyretics (Verrecchia et al., 2016). In addition, the increase in body temperature observed in FD could be associated with the alterations of heat‐dispersing mechanisms. The deposits of GL‐3 can reduce the Aδ and C fibers, affecting the reflex mechanisms of thermoregulation through the activation of muscle metabolism or cutaneous vasoconstriction (Samuelsson, Kostulas, Vrethem, Rolfs, & Press, 2014). Moreover, the endothelial dysfunction caused by GL‐3 in FD could lead to the change of vascular tone, even resulting in overt vasoconstriction phenomena (such as Raynaud phenomenon), and further impair the thermogenesis and heat dissipation (De Francesco, Mucci, Ceci, Fossati, & Rozenfeld, 2013; Satoh, 2014). Besides, the pathologically confirmed accumulation of GL‐3 in sweat glands of some patients may attribute to the lack of sweating. Taken together, these mechanisms may explain the fact that our patients all suffered from recurrent fever and hypohidrosis. Thus, fever represents a very early symptom of FD, and we suggest FD should be considered in the differential diagnosis of RFUO, especially in patients without an increment of acute phase reactants during the episodes.

It has been reported that the overlapping symptoms of FD with certain SAIDs, such as familial Mediterranean fever (FMF), made the diagnosis of FD difficult and time‐consuming. The similar clinical manifestations include RFUO, abdominal pain, arthralgia/arthritis, renal damage, and disease onset usually during childhood (Dinc et al., 2000; Lidove et al., 2012). In a study conducted on 42 patients clinically diagnosed with FMF, the S126G and M51I exonic mutations of the *GLA* gene were found in three (two harbored a single genetic alteration while one had no *MEFV* variants) (Zizzo et al., 2013). The *GLA* variants were also detected in other members of these three patients’ families, who showed some symptoms of FD upon careful analysis. Furthermore, another study demonstrated that the prevalence of FD was found as 0.56% in FMF patients from Turkey (Huzmeli et al., 2016). Interestingly, we found that two of our patients had SAIDs‐related gene variants: a maternal heterozygous *NLRC4* p.S522P, and a paternal heterozygous *NOD2* p.S30L were identified in patient 1, a paternal heterozygous *MEFV* p.R202Q was found in patient 2. They both had recurrent fever and arthralgia/arthritis, which are the common phenotypes of SAIDs and may be associated with above genes. Nevertheless, since both patients had no response to colchicine therapy, they could not be diagnosed as FMF. However, these variants might lead to more complicated manifestations, and hence a diagnostic dilemma for physicians. As reported in literature, in difficult cases due to the presence of both FD and FMF genes, characteristic storage of GL‐3 on electron microscopy in biopsy tissue is a gold standard for the diagnosis of FD (Smid et al., 2014; van der Tol et al., 2015). Therefore, we propose that for all patients presenting ambiguous symptoms of FD while concurrently carrying the *GLA* and SAIDs‐related gene variants, careful analysis of clinical manifestations and family medical histories and the use of other diagnostic tools, such as α‐Gal A enzyme activity assay, and even biopsy, should be performed to identify FD from SAIDs.

In conclusion, RFUO can be one of the initial manifestations of FD, and FD should be considered as a rare cause of RFUO. The coexistence of gene variants related to SAIDs may make the clinical phenotypes of FD more complex and prone to recurrent fever.

## CONFLICT OF INTEREST

The authors have declared no conflicts of interest.

## AUTHORS’ CONTRIBUTIONS

Di Wu and Min Shen were treating physicians for the patients and had contributed suggestions for the diagnosis and management of the patients. Yi Luo drafted the manuscript. Min Shen designed the study and revised the manuscript. All authors read and approved the final manuscript.
